# Draft Genome Sequence of a Versatile Hydrocarbon-Degrading Bacterium, *Rhodococcus pyridinivorans* Strain KG-16, Collected from Oil Fields in India

**DOI:** 10.1128/genomeA.01704-15

**Published:** 2016-02-11

**Authors:** Ramesh K. Aggarwal, Chhavi Dawar, R. Phanindranath, Lakshmi Mutnuri, Anurodh M. Dayal

**Affiliations:** a Centre for Cellular & Molecular Biology (CSIR-CCMB), Habsiguda, Hyderabad, India; b National Geophysical Research Institute (CSIR-NGRI), Habsiguda, Hyderabad, India

## Abstract

We describe here a 5.8-Mb draft genome sequence of *Rhodococcus pyridinivorans* strain KG-16, which was obtained from the soil samples collected from the oilfields of Krishna-Godavari basin in India. This genomic resource can provide insights into the pathways and mechanisms of hydrocarbon degradation and potentially aid in bioremediation applications.

## GENOME ANNOUNCEMENT

Bacterial species belonging to the genus *Rhodococcus* possess the ability to degrade a large diversity of xenobiotic and organic compounds (aliphatic, aromatic, and alicyclic hydrocarbons) that are usually present in polluted soil or oil spills (1–3). These are also suggested to be involved in the metabolism of steroids and atrazine compounds (4, 5). Strains of *Rhodococcus pyridinivorans*, like SB3094, have the ability to degrade methyl-ethyl-ketone (MEK) and thus have been used for bioaugmentation relating to the treatment of wastewater contamination by petrochemical hydrocarbons (6). Here, we report a new hydrocarbon-degrading strain, KG-16, of *R. pyridinivorans* that was isolated from 2.0-m-deep soil samples collected from the oilfields in the Krishna-Godavari (KG) basin of Andhra Pradesh, India. The species identity was confirmed by16S rRNA typing.

The draft genome sequencing of *R. pyridinivorans* KG-16 was performed using the Roche (454 FLX Titanium) pyrosequencing platform. The whole-genome sequencing (WGS) resulted in 133,886,768 bp in 327,541 reads. These reads were assembled using GS *De Novo* Assembler (version 2.8) into 87 contigs, with an *N*_50_ value of 0.35 Mb and longest contig size of 1.1 Mb. The assembled draft genome was 5.8 Mb, with a G+C content of 67.7%. Annotation was performed using the NCBI Prokaryotic Genome Annotation Pipeline (PGAP) (http://www.ncbi.nlm.nih.gov/genomes/static/Pipeline.html) and the Rapid Annotations using Subsystems Technology (RAST) server (7). KAAS (8) was used to study the metabolic pathways in the bacterium.

A total of 5,262 genes, 4,934 coding sequences (CDSs), 8 rRNAs, 51 tRNAs, and 1 noncoding RNA (ncRNA) were putatively annotated by NCBI-PGAP in the assembled draft genome. RAST annotation revealed a total of 408 subsystems, with 118 functional genes annotated in the metabolism of aromatic compound subsystems (*viz*., catabolic/degradation pathways for salicylate ester, quinate, benzoate, *p*-hydroxybenzoate, the catechol/protocatechuate branch of the beta-ketoadipate pathway, salicylate and gentisate catabolism, the homogentisate pathway, the central meta-cleavage pathway, and aromatic amine catabolism). Furthermore, in a function-based comparative analysis with the closest neighbor, *Rhodococcus jostii* RHA1, the RAST server revealed the unique presence of the catechol branch of the beta-ketoadipate pathway, the central meta-cleavage pathway of aromatic compound degradation, and aromatic amine catabolism in KG-16.

KEGG pathway analysis revealed specific genes in important pathways, like chloroalkane/alkene degradation, chlorocyclohexane and chlorobenzene degradation, metabolism of xenobiotics by cytochrome P450, synthesis and degradation of ketone bodies, ethylbenzene, atrazine, caprolactam, bisphenol, steroid, toluene, xylene, and dioxin degradation.

Many important families of microbial enzymes were annotated in KG-16, with some of the important ones being (i) lipases that can efficiently reduce hydrocarbons in contaminated soil, namely, thioesterase, carboxylesterase, lysophospholipase, and triacylglycerol lipase; (ii) peroxidases, like glutathione peroxidase, thiol-peroxidase, dye-decolorizing peroxidase, and alkyl hydroperoxidase, which degrade toxic substances; and (iii) three important monooxygenase enzymes, namely, alkanesulfonate monooxygenase, which helps in alkanesulfonate assimilation, cytochrome P450 monooxygenase, which is capable of metabolizing small-to-bulky xenobiotic compounds (9), and nitrilotriacetate monooxygenase, which initiates the biodegradation of nitrilotriacetate (10). Moreover, several tautomerases, hydroxylases, oxidoreductases, hydrolases, dehydrogenases, and transferases were also indicated in the strain. Thus, the information about the genome sequence and vast categories of important genes involved in various metabolic pathways will offer an opportunity to understand the hydrocarbon-degrading potential of the bacterium and its use in bioremediation/bioaugmentation.

### Nucleotide sequence accession number.

This whole-genome shotgun project has been deposited at DDBJ/EMBL/GenBank under the accession number AZXY00000000. The version described in this paper is the first version.
