# Correction for Aggarwal et al., “Draft Genome Sequence of a Versatile Hydrocarbon-Degrading Bacterium, Rhodococcus pyridinivorans Strain KG-16, Collected from Oil Fields in India”

**DOI:** 10.1128/MRA.01214-20

**Published:** 2020-11-05

**Authors:** Ramesh K. Aggarwal, Chhavi Dawar, R. Phanindranath, Lakshmi Mutnuri, Anurodh M. Dayal

**Affiliations:** a Centre for Cellular & Molecular Biology (CSIR-CCMB), Habsiguda, Hyderabad, India; b National Geophysical Research Institute (CSIR-NGRI), Habsiguda, Hyderabad, India; c Academy of Scientific and Innovative Research (AcSIR), Ghaziabad, India

## AUTHOR CORRECTION

Volume 4, no. 1, e01704-15, 2016, https://doi.org/10.1128/genomeA.01704-15. Page 1: The author affiliations should appear as shown in this correction.

